# Molecular evidence of *Listeria monocytogenes* infection relapse in a severe case of endocarditis

**DOI:** 10.1099/jmmcr.0.005115

**Published:** 2017-09-18

**Authors:** Giulia Ciceri, Maria Gori, Silvia Bianchi, Giovanni Corrado, Paolo Panisi, Angela Papa, Elisabetta Tanzi, Mirella Pontello

**Affiliations:** ^1^​ Department of Biomedical Sciences for Health, Università degli Studi di Milano, Milan, Italy; ^2^​ Department of Health Sciences, Università degli Studi di Milano, Milan, Italy; ^3^​ Department of Cardiology, Ospedale Valduce, Como, Italy; ^4^​ Operative Unit of Cardiac Surgery, Humanitas Gavazzeni, Bergamo, Italy; ^5^​ Operative Unit of Chemical-Clinical and Microbiological Analysis, Ospedale Valduce, Como, Italy; ^6^​ Coordinated Research Center "EpiSoMI", Università degli Studi di Milano, Milan, Italy

**Keywords:** *Listeria monocytogenes*, endocarditis, relapse, invasive listeriosis, surveillance, molecular subtyping

## Abstract

**Introduction.** Endocarditis is a rare complication of bacteraemia due to *Listeria monocytogenes* and is characterized by a high fatality rate (37–50 %). Recurrent infection by *Listeria monocytogenes* occurs even more rarely.

**Case presentation.** We report a case of recurrent *Listeria monocytogenes* infection that resulted in severe endocarditis in a 66-year-old patient with an aortic valve prosthesis. Relapse was confirmed by pulsed-field gel electrophoresis (PFGE) and multi-locus sequence typing (MLST).

**Conclusion.** Our case highlights that the molecular subtyping approach is an important tool for the detection of microbial reinfections and for the support of clinical diagnosis.

## Abbreviations

MLST, multi-locus sequence typing; PFGE, pulsed-field gel electrophoresis; ST, sequence type.

## Introduction

Listeriosis is a serious disease, with an estimated fatality rate of 20–30 %, that affects primarily pregnant women, newborns, the elderly and immunocompromised people, following ingestion of food contaminated with the Gram-positive bacterium *Listeria monocytogenes*. Common clinical manifestations include septicemia, meningitis and meningoencephalitis [1]. Endocarditis is a rare complication of bacteraemia due to *Listeria monocytogenes*, associated with a fatality rate that ranges from 37 to 50 % [2]. The clinical course usually follows a subacute pattern characterized by fever, weakness, dyspnea and cardiac murmur [2, 3]. About 60 % of listerial endocarditis is associated with valve dysfunction [3, 4], and patients with a prosthetic valve have a higher mortality rate than those with a native valve (41 and 31 %, respectively) [2, 3]. So far, approximately 80 cases of endocarditis due to *Listeria monocytogenes* have been reported in the literature [5]. Recurrent infection by *Listeria monocytogenes* occurs even more rarely. To our knowledge, in the literature only two cases confirmed clonal identity of isolates by molecular subtyping [6, 7]. We report a case of recurrent infection due to *Listeria monocytogenes* in an elderly patient with an aortic valve prosthesis, confirmed as a relapse by pulsed-field gel electrophoresis (PFGE) and by multi-locus sequence typing (MLST).

## Case report

A 66-year-old male with a history of aortic valve and ascending aorta replacement following a dissecting aneurysm of the aorta in 2010, was admitted to Sant'Anna Hospital, Como, Italy, in November 2015. He reported a two-month history of evening fever, with a maximal temperature of 38.5 °C. He denied having other clinical signs or symptoms. There was no history of smoking, alcohol abuse or illicit drug use. Blood cultures taken at the time of admission were positive for *Listeria monocytogenes.* The isolate was susceptible to ampicillin, meropenem, gentamicin and trimethoprim/sulfamethoxazole. Haematological and biochemical tests were unremarkable except for elevated C-reactive protein levels (57.9 mg l^−1^). Since the patient was allergic to penicillin, on admission he was administered oral treatment of trimethoprim/sulfamethoxazole 160/800 mg, which was continued for 10 days, three times daily. Starting from day 2, the patient was afebrile and without symptoms. On day 7, transthoracic and transesophageal echocardiograms were performed, showing an intact valve with no evidence of vegetation or endocarditis. He was discharged from the hospital in good condition on day 10, and prescribed continuation of trimethoprim/sulfamethoxazole 160/800 mg treatment for an additional 12 days, three times daily.

Six months later, in May 2016, the patient arrived at Valduce Hospital, Como, Italy, with several weeks history of evening fever, with a maximal temperature of 39 °C, not associated with any other symptoms. Again, blood cultures were positive for *Listeria monocytogenes*, displaying identical antimicrobial susceptibility as the isolates from the first episode. Upon presentation, transthoracic and transesophageal echocardiograms were performed, and endocarditis due to *Listeria monocytogenes* was diagnosed according to the blood cultures and echocardiographic imaging. Antibiotic therapy was started with meropenem 2 g intravenously, three times daily, and oral gentamicin 400 mg once a day, for six weeks. However, gentamicin was suspended on day 36, because of the onset of balance disorders. A transesophageal echocardiogram was performed again twice, on day 12 and on day 42. The first scan confirmed the presence of an echo-reflective mass and showed an intraprosthetic regurgitation, while the second scan revealed two echo-reflective masses, both with a filiform appendix. The intraprosthetic regurgitation worsened from moderate to severe. The fever rose and fell for over a month, despite antibiotics, with a maximal temperature of 38 °C. The patient became afebrile starting from day 34, and he was discharged on day 48 with instructions to continue with meropenem for another two weeks and repeat the echocardiogram, to monitor the success of treatment and evaluate the need for prosthesis removal.

In August 2016, the echocardiogram was performed, and showed a prosthetic dysfunction with a small vegetation on the ventricular surface. In November 2016, the patient was subjected to surgical replacement of his prosthetic valve at Humanitas Gavazzeni Institute, Bergamo, Italy. After an uneventful post-operative course and rehabilitation, he was discharged in good physical condition.

**Fig. 1.**
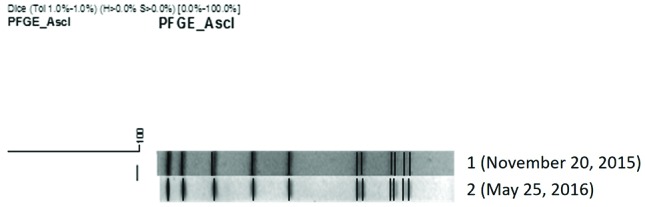
Banding profile determined by PFGE and dendrogram showing the genetic relatedness of the two isolates of *Listeria monocytogenes* recovered from the patient on November 20, 2015 and May 25, 2016.

During the surveillance of listeriosis in the Lombardy region, Italy, the two isolates of *Listeria monocytogenes* recovered from the patient’s blood cultures on 20 November 2015 and 25 May 2016 were referred to the Regional Reference Laboratory (RRL). The isolates were subtyped by using PFGE with the *Asc*I enzyme, according to the PulseNet protocol [8], and MLST, as described by Ragon *et al*. [9]. Comparison of PFGE profiles showed that the two isolates have an identical pattern (Fig. 1), and MLST indicated that they belong to the same sequence type (ST), ST288. The PFGE profile was compared with the profiles of all isolates from listeriosis cases in the RRL database (*n*=399). The comparison showed a high similarity (about 95 %) with two isolates, identified as ST288, one from Como in 2012 and one from Bergamo in 2015, the same geographical area and the same time frame of the case here described. To date, no other isolate with this ST has been recorded in the RRL database.

## Discussion

Here we report a case of a recurrent *Listeria monocytogenes* infection, which resulted in severe endocarditis. A comparison between our case and listerial endocarditis reported in the literature shows that symptoms, age of the patient and evolution of the disease observed in the present study are in line with previous studies [2–4]. Antibiotic treatment in listerial endocarditis may be efficient, even when prosthetic valves are in place, but valve replacement has to be performed when complications occur or if the organism is resistant to antibiotics [4]. In our case, the isolates showed no antibiotic resistance, but considering the recurrent listeriosis, the prosthetic dysfunction and the persistence of vegetation, surgery was necessary.

Previous case reports have documented apparent relapses of listeriosis, however molecular subtyping was not performed [10–14]; only two cases of recurrent *Listeria monocytogenes* infections with a clonally identical isolate by PFGE are reported in the literature [6, 7] (Table 1). To the best of our knowledge, this case is the first recurrent listerial infection confirmed as a relapse by two different molecular subtyping methods, PFGE and MLST. The high resolution of MLST, compared to PFGE, provides further evidence to confirm the hypothesis that the recurrent disease has been caused by a relapse with the same strain. Moreover, although PFGE is the standard typing method for *Listeria monocytogenes*, by the addition of MLST we could compare our results globally, for a better understanding of internationally occurring sequence types. To date, only 15 isolates identified as ST288 worldwide are reported in the Institut Pasteur MLST database (http://bigsdb.pasteur.fr/).

**Table 1.** Cases of recurrent listeriosis reported in the literature

**Reference**	**Clinical type of listeriosis**	**Age of the patient (years)**	**Time between episodes**	**Underlying illness**	**Outcome**	**Subtyping method(s)**	**Results**
Peetermans *et al*. [10]	Bacteraemia	46	2 years	Liver transplantation	Death	Serotyping	Different serovars
McLauchlin *et al*. [11]	Bacteraemia	8	3 months	Lymphocytic leukaemia	Favourable	Serotyping and phage typing	Identical serovar, same phage type
	Bacteraemia (first episode) and meningitis (second episode)	36	15 months	Hodgkin’s disease	Death		
	Bacteraemia	Not known	>2 years	Renal transplant	Favourable		
	Infected hip oint	59	1 year	Prosthetic hip, SLE and diabetes	Favourable		
	Bacteraemia	64	4 weeks	Lymphocytic leukaemia	Favourable		
	Meningitis	3	4 weeks	None	Favourable		
	Meningitis	57	4 months	Heart transplant	Favourable		
	Meningitis	57	9 months	Lymphocytic leukaemia and diabetes mellitus	Death	No typing information available	
	Bacteraemia (first episode) and meningitis (second episode)	25	2 weeks	Renal transplant	Favourable		
	Not known	Not known	Not known	Renal transplant	Favourable		
	Not known	Not known	Not known	Renal transplant	Favourable		
	Cerebritis	21	2 months	Renal transplant	Favourable		
	Cerebritis	27	3 weeks	Renal transplant	Favourable		
	Bacteraemia	46	19 months	Renal transplant and prosthetic hip	Favourable		
Levett *et al*. [12]	Bacteraemia	74	1 month	Chronic lymphatic leukaemia and diabetes mellitus	Death	Serotyping and RAPD	Identical serovar, same phage type, but RAPD failed to clearly demonstrate clonal identity, as no band patterns differences between the isolates and an unrelated control strain were found
Nguyen *et al*. [13]	Meningoencephalitis with septicemia	62	4 years	Heart transplant	Death	Serotyping and PFGE	Identical serovar, distinguishable PFGE patterns
Lurie *et al*. [14]	• First episode: septic miscarriage • Second episode: neonatal sepsis	37	1 year	Pregnancy	• First episode: foetal death • Second episode: favourable	No typing information available	
Sauders *et al*. [6]	Bacteraemia	68	9 months	Cancer	Favourable	Ribotyping and PFGE	Identical ribotype, indistinguishable PFGE patterns
Rohde *et al*. [7]	Bacteraemia	51	7 weeks	Aortic valve prosthesis	Favourable	RAPD and PFGE	Same phage type, indistinguishable PFGE patterns
Present case	Bacteraermia (first episode) and endocarditis (second episode)	66	6 months	Aortic valve prosthesis	Favourable	Serotyping, PFGE and MLST	Identical serovar, indistinguishable PFGE patterns, identical sequence type

Given the rarity of the strain that caused the infection and considering the time interval between the two episodes, we believe that a reinfection with the same strain was unlikely, while a persistence of the strain was more plausible. We postulate that the antibiotic treatment after the first episode was not successful, and that the relapse of infection could have arisen from the colonization of the prosthesis by *Listeria monocytogenes*, which is capable of producing biofilms. It is known from the literature that biofilms play an important role in prosthesis infection, since antibiotics cannot eradicate the bacteria within the biofilm [15]. It is plausible that the capacity of *Listeria monocytogenes* to avoid the host immune response and produce biofilm might be the cause of the relapse. Therefore, in the case of cardiopathic patients with prosthetic valve and/or pacemaker, a longer antibiotic therapy should be taken in account, also suggested by other authors [2].

In conclusion, our case highlights that the molecular subtyping approach is an important tool for the detection of microbial reinfections and for the support clinical diagnoses.
